# Supplementary material for: Modeling the C. elegans germline stem cell genetic network using automated reasoning
Source: Biosystems. 2022 Jul;217:104672. doi: 10.1016/j.biosystems.2022.104672 (PMC9142837; doi:10.1016/j.biosystems.2022.104672)
Supplement: MMC S1 — Includes information on regulation conditions, an example network toplogy, and individual cell’s setup and simulations. [file mmc1.pdf]

Supplementary Material

1. Regulation Conditions

The set of 18 regulation conditions (excluding the two threshold rules) is taken from Yordanov et al. [63]. Each column represents a different condition of a target node where a specific rule is used. The state of each target (shown as a red circle in the header diagram) is updated depending on the states of its regulators (activators or repressors). Black circles indicate whether all, some or none of the regulators are active, representing several specific conditions where a target is non-inducible and non-repressible (a), only inducible (b), only repressible (c), or both inducible and repressible (d). The conditions highlighted with yellow boxes represent non-inducible and non-repressible targets that cannot be constantly activated or repressed regardless of the state of their activators.

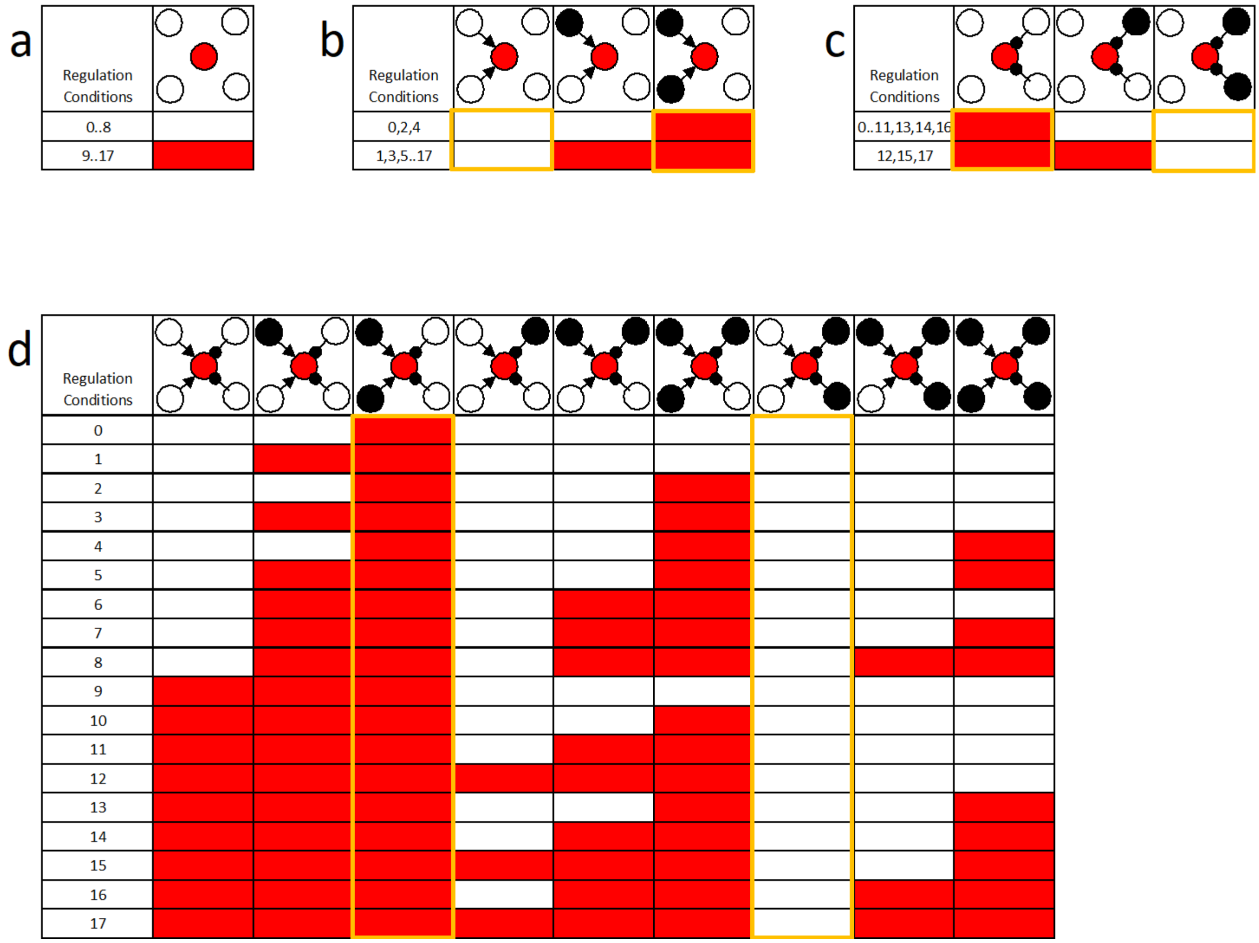

Figure S1: The set of 18 regulation conditions.

2. Example of the network topology

In Figure S2 below, an example of the network topology of a single specific solution is presented. The black colour of the component indicates that this component is knocked out in at least one specification. The blue colour of the component indicates that this component is knocked out in at least one specification and has forced expression (FE) in at least one specification. Once the solver visualizes the topology of a specific model solution, it automatically makes the *optional* interactions as red arrows that have instantiated in this solution, while the *definite* interactions remain coloured black. The *optional* interactions instantiated by the solver in this solution together with assigned regulation conditions satisfy all observations, showing how the expected behavior can be realized in this model (for details on how regulations conditions are used to determine the state of the regulated component, see [63] and Figure S1).

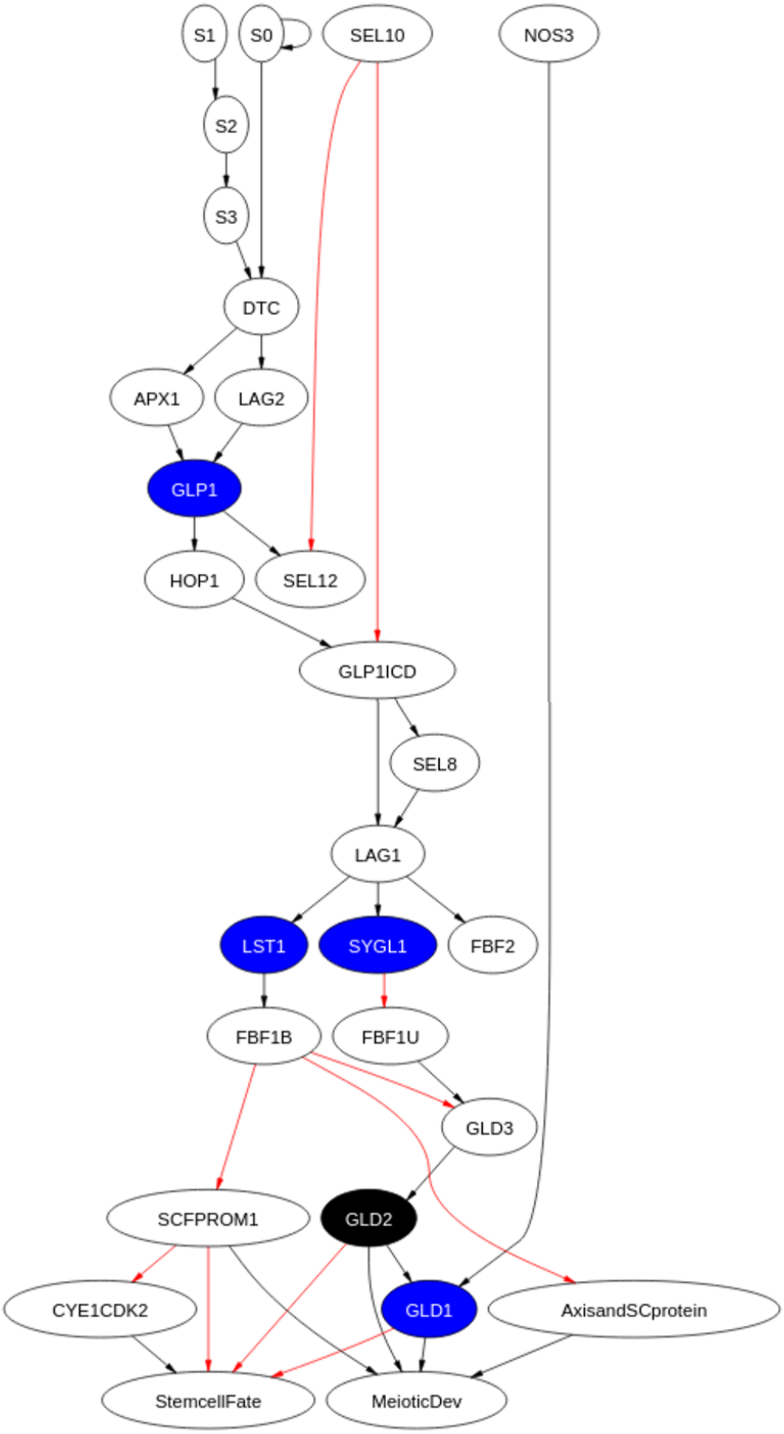

Figure S2: Visualization of the topology of a specific model solution.

3. Individual cell's setup

This setup allows to simulate a stem cell that loses DTC signaling as it moves from the distal-most end of the germ line further proximally. Therefore, the specified initial state of the simulation along with DTC signal is *StemcellFate* and the expected outcome in this example after 20 steps is meiotic development *MeioticDev*. To model degrading DTC signaling (from present to absent) inputs were separated into 3 generic signals that we call signal 1, 2, and 3 (S1, S2, S3) and that are connected in series (see Figure S1) resulting in the absence of DTC signal from $t = 4$ onwards. The initiation of the network interactions starts when the DTC signals to the germ line by expressing LAG-2 and APX-1 proteins [48], [31] at time step $t = 1$. APX-1 and LAG-2 redundantly activate GLP-1 Notch receptor, which results in the release of the GLP-1 intracellular domain [23]. Other GLP-1 signaling pathway genes *hop-1* and *sel-12* are activated at $t = 3$ of the simulation. The network state at $t = 4$ captures the formation of a ternary complex containing GLP1ICD, LAG-1, and SEL-8 [50] (see Figure S2). At the next steps, GLP-1 signaling leads to the activation of *lst-1* and *sygl-1* genes and thereby to the inhibition of the GLD-1, GLD-2 and SCF^{PROM-1} meiotic entry pathways through FBF. Once FBF is inactivated, meiotic entry pathways return to being active and *MeioticDev* is constituted from time step $t = 15$.

step	S1	S2	S3	DTC	StemcellFate	APX1	LAG2	GLP1	HOP1	SEL12	SEL10	GLP1ICD	SEL8	LAG1	SYGL1	LST1	FBF1U	FBF1B	FBF2	GLD3	AxisandSCprotein	SCFPROM1	GLD2	NOS3	CYE1CDK2	GLD1	MeioticDev
0	true	true	true	true	true	false	false	false	false	false	false	false	false	false	true	false	false	true	false	false	false	false	false	false	true	false	false
1	false	true	true	true	true	true	true	false	false	false	false	false	false	false	false	false	false	true	false	false	false	false	false	false	true	false	false
2	false	false	true	true	true	true	true	true	false	false	false	false	false	false	false	false	true	false	false	false	false	false	false	false	true	false	false
3	false	false	false	true	true	true	true	true	true	true	false	false	false	false	false	false	true	false	false	true	true	true	false	false	true	false	false
4	false	false	false	false	true	true	true	true	true	true	false	true	false	false	false	false	true	false	false	true	true	true	true	false	false	false	false
5	false	false	false	false	false	false	false	true	true	true	false	true	true	false	false	false	true	false	false	true	true	true	true	false	false	true	false
6	false	false	false	false	false	false	false	false	true	true	false	true	true	true	false	false	true	false	false	true	true	true	true	false	false	true	true
7	false	false	false	false	false	false	false	false	false	false	false	true	true	true	true	true	true	false	true	true	true	true	true	false	false	true	true
8	false	false	false	false	false	false	false	false	false	false	false	false	true	true	true	true	false	true	true	true	true	true	true	false	false	true	true
9	false	false	false	false	false	false	false	false	false	false	false	false	false	false	true	true	false	true	true	false	false	false	true	false	false	true	true
10	false	false	false	false	false	false	false	false	false	false	false	false	false	false	false	false	false	true	false	false	false	false	false	false	true	true	false
11	false	false	false	false	true	false	false	false	false	false	false	false	false	false	false	false	true	false	false	false	false	false	false	false	true	false	false
12	false	false	false	false	true	false	false	false	false	false	false	false	false	false	false	false	true	false	false	true	true	true	false	false	true	false	false
13	false	false	false	false	true	false	false	false	false	false	false	false	false	false	false	false	true	false	false	true	true	true	true	false	false	false	false
14	false	false	false	false	false	false	false	false	false	false	false	false	false	false	false	false	true	false	false	true	true	true	true	false	false	true	false
15	false	false	false	false	false	false	false	false	false	false	false	false	false	false	false	false	true	false	false	true	true	true	true	false	false	true	true
16	false	false	false	false	false	false	false	false	false	false	false	false	false	false	false	false	true	false	false	true	true	true	true	false	false	true	true
17	false	false	false	false	false	false	false	false	false	false	false	false	false	false	false	false	true	false	false	true	true	true	true	false	false	true	true
18	false	false	false	false	false	false	false	false	false	false	false	false	false	false	false	false	true	false	false	true	true	true	true	false	false	true	true
19	false	false	false	false	false	false	false	false	false	false	false	false	false	false	false	false	true	false	false	true	true	true	true	false	false	true	true
20	false	false	false	false	false	false	false	false	false	false	false	false	false	false	false	false	true	false	false	true	true	true	true	false	false	true	true

Figure S3: The table of Boolean values of each gene simulating a stem cell that loses DTC signaling.

Finally, we simulated a cell further from the DTC in the proliferative zone by reducing but not eliminating the DTC signal duration (DTC = 1 at $t = 0$ only). The analysis of these simulations showed that a reduction of the DTC signal is not enough to ensure that the cell always undergoes meiotic development. While in some simulations the cell enters meiotic development state (Figure S4), in others it exhibits transient recurrence of stem cell fate (Figure S5).

step	DTC	StemcellFate	APX1	LAG2	GLP1	HOP1	SEL12	SEL10	GLP1ICD	SEL8	LAG1	SYGL1	LST1	FBF1U	FBF1B	FBF2	GLD3	AxisandSCprotein	SCFPROM1	GLD2	NOS3	CYE1CDK2	GLD1	MeioticDev
0	true	false	true	true	true	false	false	false	false	false	false	false	false	true	false	false	false	false	false	false	false	true	false	false
1	false	true	true	true	true	true	true	true	false	false	false	false	false	true	false	false	true	true	true	false	false	true	false	false
2	false	false	false	false	true	true	true	true	true	false	false	false	false	true	false	false	true	true	true	true	false	false	false	false
3	false	false	false	false	false	true	true	true	true	true	false	false	false	true	false	false	true	true	true	true	false	false	true	false
4	false	false	false	false	false	false	false	true	true	true	true	false	false	true	false	false	true	true	true	true	false	false	true	true
5	false	false	false	false	false	false	false	true	false	true	true	true	false	true	false	true	true	true	true	true	false	false	true	true
6	false	false	false	false	false	false	false	true	false	false	false	true	false	false	true	true	true	true	true	true	false	false	true	true
7	false	false	false	false	false	false	false	true	false	false	false	false	false	false	true	false	false	false	false	true	false	false	true	true
8	false	false	false	false	false	false	false	true	false	false	false	false	false	true	false	false	false	true	false	false	false	true	true	false
9	false	false	false	false	false	false	false	true	false	false	false	false	false	true	false	false	true	true	true	false	false	true	false	false
10	false	false	false	false	false	false	false	true	false	false	false	false	false	true	false	false	true	true	true	true	false	false	false	false
11	false	false	false	false	false	false	false	true	false	false	false	false	false	true	false	false	true	true	true	true	false	false	true	false
12	false	false	false	false	false	false	false	true	false	false	false	false	false	true	false	false	true	true	true	true	false	false	true	true
13	false	false	false	false	false	false	false	true	false	false	false	false	false	true	false	false	true	true	true	true	false	false	true	true
14	false	false	false	false	false	false	false	true	false	false	false	false	false	true	false	false	true	true	true	true	false	false	true	true
15	false	false	false	false	false	false	false	true	false	false	false	false	false	true	false	false	true	true	true	true	false	false	true	true
16	false	false	false	false	false	false	false	true	false	false	false	false	false	true	false	false	true	true	true	true	false	false	true	true
17	false	false	false	false	false	false	false	true	false	false	false	false	false	true	false	false	true	true	true	true	false	false	true	true
18	false	false	false	false	false	false	false	true	false	false	false	false	false	true	false	false	true	true	true	true	false	false	true	true
19	false	false	false	false	false	false	false	true	false	false	false	false	false	true	false	false	true	true	true	true	false	false	true	true
20	false	false	false	false	false	false	false	true	false	false	false	false	false	true	false	false	true	true	true	true	false	false	true	true

Figure S4: Simulation of a stem cell that is out of range of the DTC signal – Experiment No.1.

step	DTC	StemcellFate	APX1	LAG2	GLP1	HOP1	SEL12	SEL10	GLP1ICD	SEL8	LAG1	SYGL1	LST1	FBF1U	FBF1B	FBF2	GLD3	AxisandSCprotein	SCFPROM1	GLD2	NOS3	CYE1CDK2	GLD1	MeioticDev
0	true	false	true	true	true	false	false	false	false	false	false	false	false	true	false	false	false	false	false	false	false	true	false	false
1	false	true	true	true	true	true	true	false	false	false	false	false	false	true	false	false	true	true	true	false	false	true	true	false
2	false	true	false	false	true	true	true	false	true	false	false	false	false	true	false	false	true	true	true	true	false	false	true	false
3	false	false	false	false	false	true	true	false	true	true	true	false	false	true	false	false	true	true	true	true	false	false	true	true
4	false	false	false	false	false	false	false	false	true	true	true	true	false	true	false	true	true	true	true	true	false	false	true	true
5	false	false	false	false	false	false	false	false	false	true	true	true	false	true	true	true	true	false	true	true	false	false	false	true
6	false	false	false	false	false	false	false	false	false	false	true	true	false	true	true	true	false	false	false	true	false	false	false	false
7	false	false	false	false	false	false	false	false	false	false	false	true	false	true	true	true	false	false	false	false	false	true	false	false
8	false	true	false	false	false	false	false	false	false	false	false	false	false	true	true	false	false	false	false	false	false	true	false	false
9	false	true	false	false	false	false	false	false	false	false	false	false	false	true	false	false	false	true	false	false	false	true	false	false
10	false	true	false	false	false	false	false	false	false	false	false	false	false	true	false	false	true	true	true	false	false	true	true	false
11	false	true	false	false	false	false	false	false	false	false	false	false	false	true	false	false	true	true	true	true	false	false	true	false
12	false	false	false	false	false	false	false	false	false	false	false	false	false	true	false	false	true	true	true	true	false	false	true	true
13	false	false	false	false	false	false	false	false	false	false	false	false	false	true	false	false	true	true	true	true	false	false	true	true
14	false	false	false	false	false	false	false	false	false	false	false	false	false	true	false	false	true	true	true	true	false	false	true	true
15	false	false	false	false	false	false	false	false	false	false	false	false	false	true	false	false	true	true	true	true	false	false	true	true
16	false	false	false	false	false	false	false	false	false	false	false	false	false	true	false	false	true	true	true	true	false	false	true	true
17	false	false	false	false	false	false	false	false	false	false	false	false	false	true	false	false	true	true	true	true	false	false	true	true
18	false	false	false	false	false	false	false	false	false	false	false	false	false	true	false	false	true	true	true	true	false	false	true	true
19	false	false	false	false	false	false	false	false	false	false	false	false	false	true	false	false	true	true	true	true	false	false	true	true
20	false	false	false	false	false	false	false	false	false	false	false	false	false	true	false	false	true	true	true	true	false	false	true	true

Figure S5: Simulation of a stem cell that is out of range of the DTC signal – Experiment No.2.

Next, we checked whether some network component promotes stem cell fate, despite a reduced level of DTC signaling. We found that CYE1/CDK2 (cyclin E and cyclin-dependent kinase) appears to be an active regulator of the stem cell fate. Therefore, we specified external selfdegrading signal, which reduces CYE1/CDK2 activity for realistic simulation of this scenario (Figure S6). This is consistent with investigation by Fox et al. [20], which showed that CYE1/CDK2 plays important role in promoting the proliferative fate, specifically in cells with reduced GLP-1 signaling.

step	DTC	StemcellFate	APX1	LAG2	GLP1	HOP1	SEL12	SEL10	GLP1ICD	SEL8	LAG1	SYGL1	LST1	FBF1U	FBF1B	FBF2	GLD3	AxisandSCprotein	SCFPROM1	GLD2	NOS3	S	CYE1CDK2	GLD1	MeioticDev
0	true	false	true	true	true	false	false	false	false	false	false	false	false	true	false	false	false	false	false	false	false	true	true	false	false
1	false	true	true	true	true	true	true	false	false	false	false	false	false	true	false	false	true	true	true	false	true	false	true	false	false
2	false	true	false	false	true	true	true	false	true	false	false	false	false	true	false	false	true	true	true	true	true	false	false	true	false
3	false	false	false	false	false	true	true	false	true	true	true	false	false	true	false	false	true	true	true	true	true	false	false	true	true
4	false	false	false	false	false	false	true	false	true	true	true	true	true	true	false	true	true	true	true	true	true	false	false	true	true
5	false	false	false	false	false	false	true	false	false	true	true	true	true	true	true	true	true	true	true	true	true	false	false	true	true
6	false	false	false	false	false	false	true	false	false	false	true	true	true	true	true	true	true	false	true	true	true	false	false	true	true
7	false	false	false	false	false	false	true	false	false	false	false	true	true	true	true	true	true	false	true	true	true	false	false	true	false
8	false	false	false	false	false	false	true	false	false	false	false	false	false	true	true	false	true	false	true	true	true	false	false	true	false
9	false	false	false	false	false	false	true	false	false	false	false	false	false	true	false	false	true	false	true	true	true	false	false	true	false
10	false	false	false	false	false	false	true	false	false	false	false	false	false	true	false	false	true	true	true	true	true	false	false	true	false
11	false	false	false	false	false	false	true	false	false	false	false	false	false	true	false	false	true	true	true	true	true	false	false	true	true
12	false	false	false	false	false	false	true	false	false	false	false	false	false	true	false	false	true	true	true	true	true	false	false	true	true
13	false	false	false	false	false	false	true	false	false	false	false	false	false	true	false	false	true	true	true	true	true	false	false	true	true
14	false	false	false	false	false	false	true	false	false	false	false	false	false	true	false	false	true	true	true	true	true	false	false	true	true
15	false	false	false	false	false	false	true	false	false	false	false	false	false	true	false	false	true	true	true	true	true	false	false	true	true
16	false	false	false	false	false	false	true	false	false	false	false	false	false	true	false	false	true	true	true	true	true	false	false	true	true
17	false	false	false	false	false	false	true	false	false	false	false	false	false	true	false	false	true	true	true	true	true	false	false	true	true
18	false	false	false	false	false	false	true	false	false	false	false	false	false	true	false	false	true	true	true	true	true	false	false	true	true
19	false	false	false	false	false	false	true	false	false	false	false	false	false	true	false	false	true	true	true	true	true	false	false	true	true
20	false	false	false	false	false	false	true	false	false	false	false	false	false	true	false	false	true	true	true	true	true	false	false	true	true

Figure S6: The table of Boolean values of each gene simulating a stem cell that is out of range of the DTC signal.
